# Conflicting evidence for the use of caudal autotomy in mesosaurs

**DOI:** 10.1038/s41598-020-63625-0

**Published:** 2020-04-28

**Authors:** Mark J. MacDougall, Antoine Verrière, Tanja Wintrich, Aaron R. H. LeBlanc, Vincent Fernandez, Jörg Fröbisch

**Affiliations:** 1 Museum für Naturkunde, Leibniz-Institut für Evolutions- und Biodiversitätsforschung, Berlin, Germany; 2 Rheinische Friedrich-Wilhelms-Universität Bonn, Bonn, Germany; 3 University of Alberta, Edmonton, Canada; 4 Natural History Museum London, London, UK; 5 European Synchrotron Radiation Facility, Grenoble, France; 6 Humboldt-Universität zu Berlin, Berlin, Germany

**Keywords:** Evolution, Palaeontology

## Abstract

The early Permian mesosaurs were the first amniotes to re-invade aquatic environments. One of their most controversial and puzzling features is their distinctive caudal anatomy, which has been suggested as a mechanism to facilitate caudal autotomy. Several researchers have described putative fracture planes in mesosaur caudal vertebrae — unossified regions in the middle of caudal vertebral centra — that in many extant squamates allow the tail to separate and the animal to escape predation. However, the reports of fracture planes in mesosaurs have never been closely investigated beyond preliminary descriptions, which has prompted scepticism. Here, using numerous vertebral series, histology, and X-ray computed tomography, we provide a detailed account of fracture planes in all three species of mesosaurs. Given the importance of the tail for propulsion in many other aquatic reptiles, the identification of fracture planes in mesosaurs has important implications for their aquatic locomotion. Despite mesosaurs apparently having the ability to autotomize their tail, it is unlikely that they actually made use of this behaviour due to a lack of predation pressure and no record of autotomized tails in articulated specimens. We suggest that the presence of fracture planes in mesosaurs is an evolutionary relic and could represent a synapomorphy for an as-yet undetermined terrestrial clade of Palaeozoic amniotes that includes the earliest radiation of secondarily aquatic tetrapods.

## Introduction

As the first group of amniotes to return to an aquatic lifestyle, and a key line of evidence for the theory of continental drift^1^, mesosaurs have figured prominently in reconstructions of early amniote evolution. Mesosauridae is currently composed of three monotypic genera (*Mesosaurus tenuidens* Gervais, 1865^2^, *Stereosternum tumidum* Cope, 1886^3^, and *Brazilosaurus sanpauloensis* Shikama and Ozaki, 1966^4^), all of which are only known from localities that would have been part of an inland Gondwanan sea during the early Permian. Despite debate regarding the exact placement of Mesosauridae among early reptiles, researchers generally agree that they represent one of the most basal reptile clades^5–9^.

The anatomy of mesosaurs has also been debated extensively, and one of the more contentious aspects concerns whether or not they had the capacity for caudal autotomy. Caudal autotomy is the ability to drop a part of the tail in order to escape predation, an anti-predator behaviour that is prevalent in several clades of extant lepidosaurs^10,11^. In extant reptiles, caudal autotomy can occur between caudal vertebrae (intervertebral) or along planes of weakness along the caudal centrum that split a vertebra in two (intravertebral). The latter form of autotomy is the most common among extant reptiles and is the only traceable form of autotomy in the fossil record^12,13^. Regeneration of a cartilage cone posterior to the autotomized region may also occur following autotomy, however, several lineages of lepidosaurs do not regenerate their tails^11^.

Several researchers have briefly mentioned the presence of fracture planes on the centra of mesosaur caudal vertebrae, suggesting that mesosaurs may have exhibited intravertebral autotomy^14–16^. However, these observations were questioned by Modesto^17^, who suggested that these fracture planes were a misinterpretation, and were instead the result of postmortem breakage. Recently, by building upon earlier observations^12,18^ and using modern histological techniques, LeBlanc *et al*.^13^ argued for the presence of caudal autotomy in early Permian captorhinids by demonstrating the presence of fracture planes along their caudal vertebrae. LeBlanc *et al*.^13^ followed Modesto’s^17^ interpretation and concluded that captorhinids were thus the earliest and only confirmed record of caudal autotomy in Palaeozoic amniotes.

Despite the contentions over caudal autotomy in mesosaurs, the presence of fracture planes in mesosaur specimens has never been thoroughly examined beyond preliminary reports and illustrations. Using X-ray micro computed tomography (µCT), histological thin sections, and high-resolution photographs, we provide the first detailed account of fracture planes along the caudal vertebrae of several mesosaur specimens and demonstrate that mesosaurs would have been theoretically capable of caudal autotomy, but likely did not utilize the behaviour. Furthermore, we address the implications for the aquatic lifestyle of mesosaurs and the way they would have propelled themselves through the water, given the potential capacity to drop their tails.

**Institutional abbreviations: IGPB** – Institut für Geowissenschaften, Paläontologie, Universität Bonn, Germany; **NHMW** – Naturhistorisches Museum Wien, Austria; **NRM** – Naturhistorika riksmuseet, Stockholm, Sweden; **PIMUZ** – Paläontologisches Institut und Museum der Universität Zürich, Switzerland; **SMNK** – Staatliches Museum für Naturkunde Karlsruhe, Germany; **SMNS** – Staatliches Museum für Naturkunde Stuttgart, Germany.

## Results

### Presence of fracture planes

**Figure 1:**
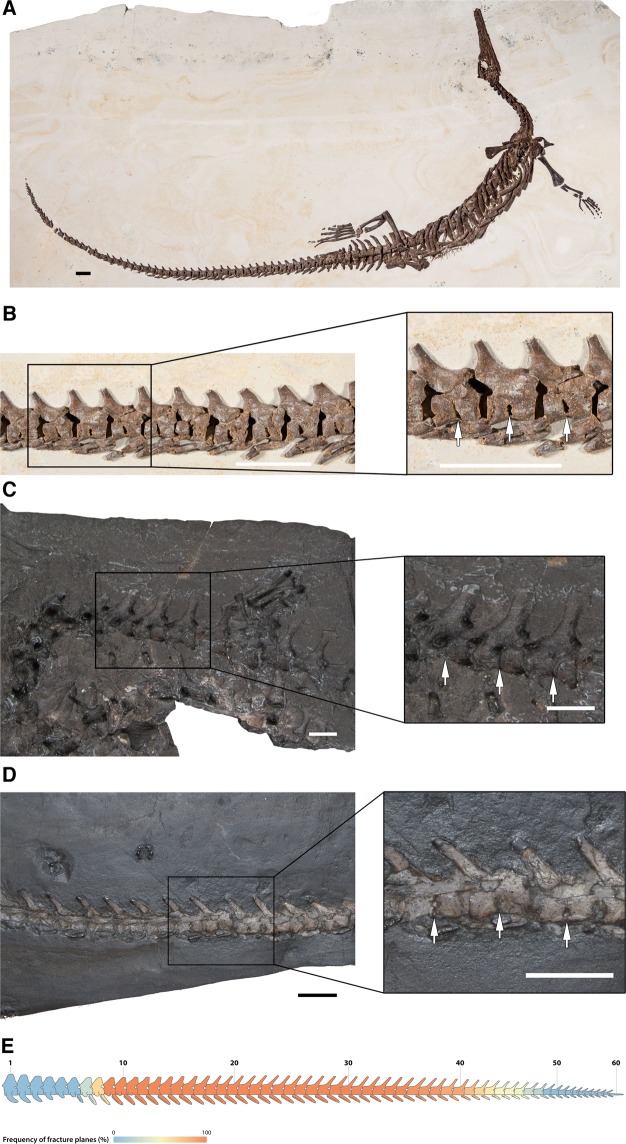
Examples of fracture planes in various mesosaur taxa. (**A**) Complete skeleton of *Stereosternum* (PIMUZ A III 591); fracture planes on caudals of (**B)**, *Stereosternum* (PIMUZ A III 591), (**C)** *Mesosaurus* (NRM PZ R 640), and (**D)**, *Brazilosaurus* (SMNS 51560). Arrows indicate the position of fracture planes. (**E)** Heat map obtained from 42 mesosaur specimens indicating which caudal vertebrae exhibit fracture planes. All Scale bars equal 1 cm.

**Figure 2:**
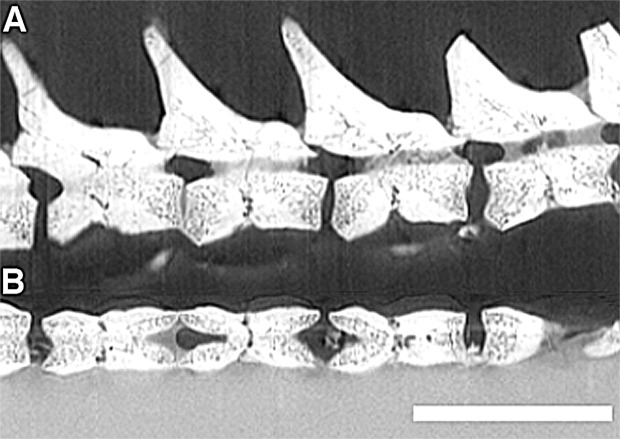
Micro computed tomography data of *Stereosternum*, PIMUZ A III 591, showing virtual sections through part of the caudal series in (**A)**, sagittal and (**B)**, transverse planes. Scale bar equals 4 mm.

**Figure 3:**
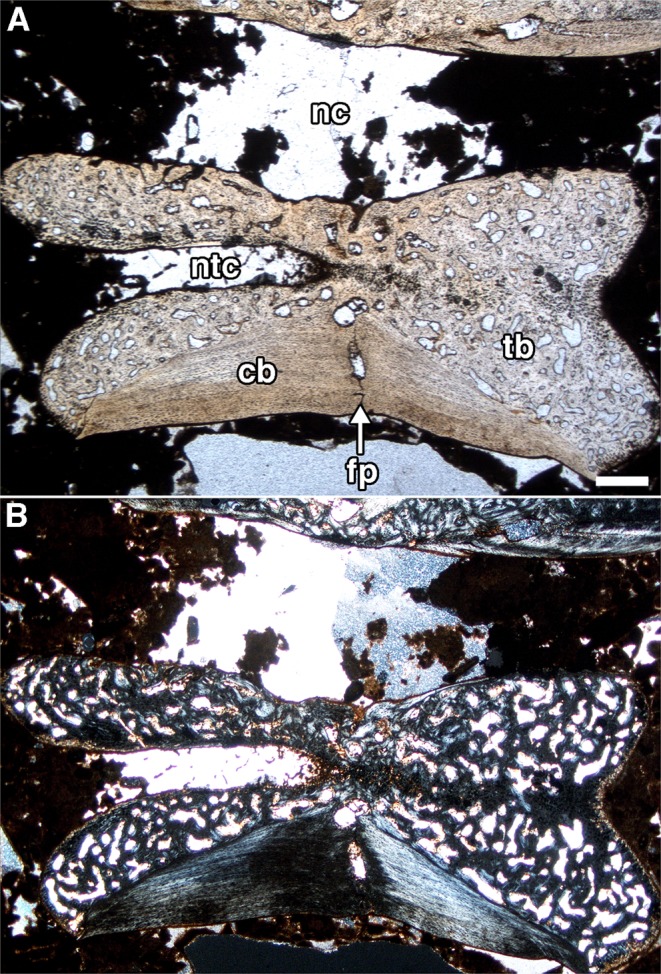
Sagittal section through the centrum of an isolated mesosaur caudal vertebrae with fracture plane (IGPB R 624) under (**A)**, normal, and (**B)**, cross polarized light. Abbreviations: **cb**, cortical bone; **fp**, fracture plane; **nc**, neural canal; **ntc**, notochordal canal; **tb**, trabecular bone. Scale bar equals 500 *μ*m.

Our survey of several articulated mesosaur specimens revealed that the large majority of caudal vertebrae possess dorsoventral splits along the midlines of the ventral half of the centra. Despite interpretations in the past that these transverse splits were taphonomic^17^, this clearly cannot be the case, because: (1) they are restricted to the caudal centra; (2) they are present in all three mesosaur taxa (Fig. 1); and (3) they occur in multiple well preserved specimens (Fig. 1, Sup. Info.). Furthermore, the extent and form of the observed splits is largely consistent with the fracture planes that have been previously identified in the Palaeozoic reptile *Captorhinus*^13^, though there are some differences. The fracture planes are characterized by an unossified region that extends dorsally from the ventral edge of the caudal centrum to the base of the neural spine, positioned at the dorsoventrally constricted portion of the centrum (Fig. 2). A histological thin section through the sagittal plane of an isolated mesosaur caudal vertebra with a fracture plane (Fig. 3) confirms what we observed externally and in the µCT data. As in *Captorhinus*^13^, the bone that surrounds the fracture plane is compact cortical bone, which is thickest at the ventral portion of the centrum (Fig. 3). This region of cortical bone is made up of several concentric, poorly vascularized layers, and overall is much thicker than what is observed in *Captorhinus*, which is consistent with pachyostosis in some aquatic tetrapods^19^. Furthermore, the fracture plane is slightly shorter and does not appear to enter the neural canal as in *Captorhinus*. Highly vascularized endochondral bone forms much of the rest of the centrum.

The fracture planes in *Stereosternum* start on the eighth caudal vertebra and continue along subsequent caudals to around the 40^th^ caudal vertebra, only disappearing towards the end of the tail. Based on the material used in this study it is unclear if the fracture planes would have started on the same caudal vertebra in *Mesosaurus* and *Brazilosaurus*, as the caudal on which fracture planes start can vary even in extant taxa^10,13,20^. Despite the presence of fracture planes in mesosaur caudal vertebrae, it is unclear if mesosaurs exhibited caudal autotomy in life. In articulated specimens where part of the tail is missing, the centra are either not clearly exposed or the tail is cut off between individual vertebrae at the edge of the slab on which a specimen is preserved (pers. obs. MJM and AV).

### Presence of webbing on the limbs of mesosaurs

**Figure 4:**
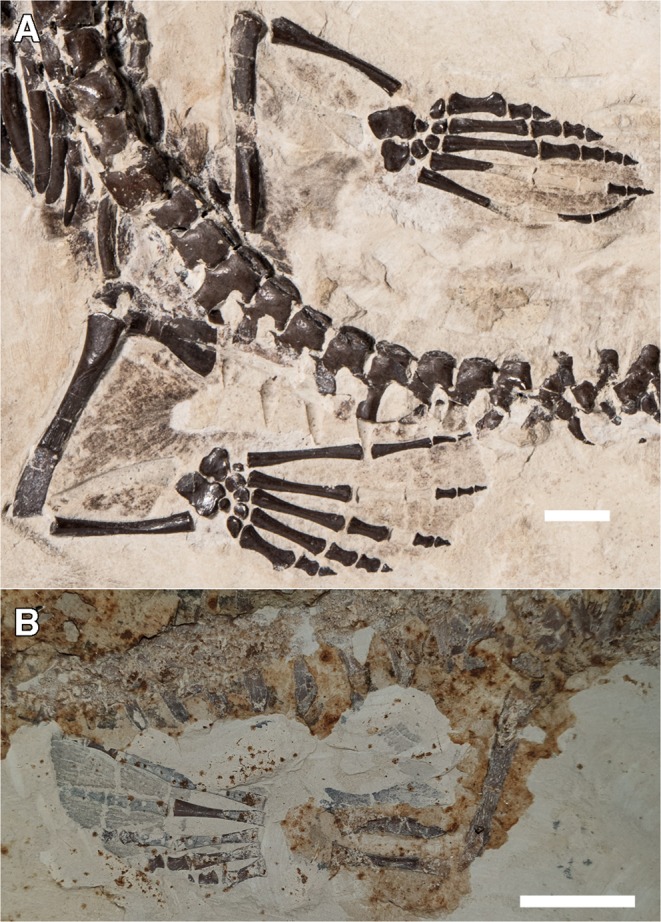
Webbing impression preserved on mesosaur specimens. (**A)**, hindlimb of *Stereosternum*, SMNK PAL 3806, and (**B)**, hindlimb of *Stereosternum*, URCR 64. Scale bars equals 1 cm.

We also assessed the potential swimming mode in mesosaurs in order to better understand aquatic locomotion in an aquatic amniote that could potentially drop its tail. In all three mesosaurid species, the pes acquires an elongated paddle shape, and interdigit webbing is prominently preserved on the pes of some specimens of *Stereosternum* (Fig. 4). The webbing observed on these specimens is visible as an impression between the digits of the hindlimbs, being clearly visible between the digits that are spread apart. Furthermore, on SMNK PAL 3806 there is also tissue impression associated with the rest of the hindlimbs, which is best observed on the left hindlimb of the specimen. This tissue impression adds considerable mass to the hindlimbs, suggesting that they were used extensively during locomotion.

## Discussion

### The evolutionary and behavioural implications of caudal autotomy in mesosaurs

Given the presence of fracture planes on the caudal centra of all three mesosaur genera, these early amniotes had the potential to drop their tails if grabbed by a predator, similar to captorhinids^13^. The presence of fracture planes on the caudal centra of mesosaurs also has important implications for the evolutionary history of caudal autotomy in amniotes.

Mesosauridae are historically considered either as basal-most parareptiles^6,9^, or basal-most reptiles^5,8,21^. The presence of fracture planes on the caudal vertebrae in captorhinids, and now mesosaurs, suggests that caudal autotomy could potentially be an ancestral feature of reptiles or an even more inclusive group, however, more evidence of this structure is needed in other taxa before any kind of conclusions can be confidently made. Currently, the potential for caudal autotomy has never been identified in synapsids, the other main lineage of amniotes. Outside of mesosaurs fracture planes have not been identified in other parareptiles^13^, but they have been identified in ‘microsaurs’, another group of Palaeozoic fossil tetrapods, specifically in the taxon *Microbrachis*^22^.

Despite the presence of fracture planes in mesosaur caudal vertebra, it is important to note that as far as we are aware there has never been a mesosaur specimen found in which its tail has autotomized. The first Palaeozoic reptile for which caudal autotomy was confirmed was the early reptile *Captorhinus*^12,13,18^, with caudal fracture planes being identified in partial caudal skeletons of at least two species. However, unlike in *Captorhinus*, mesosaur specimens are often preserved with complete tails, providing better preservational data about how frequent or infrequent the behaviour was. The absence of autotomized tails indicates that this was either a very rare occurrence that is difficult to capture in the fossil record, or that mesosaurs may not have actually used this escape behaviour, despite retaining the ability to do so. By comparison, some articulated fossil lepidosaurs preserve autotomized and even regenerated tails^23^, but these types of specimens are absent from the mesosaur fossil record, despite the large number of identified specimens with articulated caudal skeletons. Furthermore, regeneration of a cartilaginous cone does not always follow autotomy in several groups of extant lepidosaurs^11^. Therefore, a lack of regenerated tails in the fossil record does not necessarily indicate the absence of tail autotomy in a taxon.

All three currently known mesosaur species are known from early Permian localities that were once part of the Whitehill-Irati Sea, a shallow body of water in northern Gondwana^24^, mesosaurs would have represented the largest animal in this sea, and they did not have any known predators. Stratagraphically, the Irati Formation is divided into two distinct members, the lower Taquaral Member and the higher Assistência Member^25,26^ Though relatively large chondrichthyans are present they are not found in the same strata as mesosaurs^27^, with mesosaurs being found in the upper Assistência Member^25^. The only taxa known from the Assistência Member are mesosaurs, paleonisciform fish, pygocephalomorph crustaceans, ostracods, brachiopods, foraminiferans, and sponges^24,25,27,28^. The potential lack of predation pressure in this aquatic environment suggests that mesosaurs may not have autotomized their tails, as this behaviour is primarily used in other taxa to escape predators^10,11,20,29^.

Alternatively, mesosaurs may not have autotomized their tails because of their importance in aquatic locomotion^30^. Furthermore, our histological data show that the fracture planes present in *Stereosternum* are surrounded by a very thick layer of cortical bone, and the fracture plane itself does not enter the neural canal (Figs. 2, 3) as it does in *Captorhinus*^13^. Such features would tend to reinforce the vertebrae and make them less susceptible to breaking along the planes in mesosaurs. This suggests that the ability to drop their tail was limited or that the presence of intravertebral fracture planes represents the ancestral condition for a more inclusive, terrestrial clade of early amniotes. Some of these taxa would have made use of the behaviour, whereas others would not have (e.g. mesosaurs), the driving factor being the predator-prey interactions in the communities in which they lived, and their trophic niches within those communities.

However, mesosaurs may have had the ability to occasionally come up onto land^31^, and the ability to escape potential terrestrial predators using caudal autotomy in order to return to the water would have been beneficial. Furthermore, it has been suggested that mesosaurs may have been cannibalistic, with adults preying on juveniles^32^. If this was the case, the ability to autotomize the tail would have been beneficial for younger mesosaurs, increasing their chances to avoid predation by older individuals. However, this hypothesis of juvenile cannibalism lacks convincing evidence^28^.

### The implications of caudal autotomy for aquatic locomotion in mesosaurs

The presence of fracture planes in mesosaurs also raises questions regarding how they would have moved through the aquatic medium in which they spent most of their lives. While it has never been thoroughly investigated and little is known about their swimming habits, it has been suggested that mesosaurs could have used a sub-anguilliform (axial undulations that involve most of the body length, but especially the tail) mode of swimming^30,33^. If mesosaurs could autotomize their tails, this would have significantly affected their swimming capabilities and ability to catch prey^34^. The presence of autotomous caudal vertebrae in mesosaurs therefore leads us to question how important the tail was in propelling them through the water. There are two main possibilities regarding the usage of their tail in aquatic locomotion, the first being that if mesosaurs were autotomizing their tails the limbs would likely play a larger role in swimming to compensate for a reduced tail. The second possibility is that, despite having the potential to autotomize their tails, the fracture planes were evolutionary relics, the behaviour was not actually used, and they were tail-driven swimmers as has been previously suggested.

The tails of mesosaurs do not exhibit many of the adaptations for tail driven swimming observed in other secondarily aquatic tetrapods. For example, in the case of the early mosasauroid *Vallecillosaurus*, most caudal vertebrae possess tall neural spines and haemal arches, with the exception of shorter neural spines on the anteriormost caudals^35^. Mesosaurs lack enlarged neural spines on the caudals, at least to the degree observed in *Vallecillosaurus* and other mosasauroids.

Aside from mosasaurs, there are other secondarily aquatic squamates worth comparing to mesosaurs, such as the Cretaceous marine lizard *Pontosaurus*, which also exhibits several adaptations for tail-driven locomotion^36^. These include the presence of very long tails that make up 2/3 of overall body length, and the tail being dorsoventrally deepened for much of its length^36^, both of which are characteristics not observed in mesosaurs.

The Triassic Nothosauroidea (pachypleurosaurs and nothosaurs) represent another group of aquatic reptiles that is quite well studied. The tail of pachypleurosaur nothosauroids is about the same length as the rest of the body and is not particularly dorsoventrally expanded^37^. Interestingly, nothosaurs are interpreted as utilizing both their tail and webbed limbs during aquatic locomotion^38^. The overall tail and limb morphology of nothosaurs is similar to what is observed for mesosaurs, which may suggest that mesosaurs may have also employed a type of locomotion similar to nothosaurs. Furthermore, it has been inferred that most nothosauroids would have been restricted to near-shore, shallow marine environments^39,40^, which is very similar to the environment mesosaurs are considered to have occupied^24,30^.

The Late Jurassic rhynchocephalian *Vadasaurus* is interpreted as a semiaquatic reptile that also possesses fracture planes^41^. Much like mesosaurs, there is currently no evidence that *Vadasaurus* would have employed caudal autotomy, despite retaining the anatomical structures to do so. This suggests that the situation is similar to what is observed in mesosaurs, where the fracture planes are plesiomorphic features of a larger clade.

Mesosaurs have also been compared to the marine iguana *Amblyrhynchus*^30^, as it is one of the few extant, limbed reptiles that exhibits a similar, semi-aquatic lifestyle. *Amblyrhynchus* utilizes an anguilliform mode of swimming, and is one of the few iguanids that does not exhibit caudal autotomy^42,43^, meaning that one of the main structures it uses for propulsion will always be present. This suggests it may not be the best analogue for swimming in mesosaurs. Furthermore, we showed here that some mesosaur specimens have impressions of prominent and extensive webbing between the digits of their autopodia (Fig. 4), whereas the autopodia of *Amblyrhynchus* do not exhibit such webbing^44^. This, combined with the paddle-like limbs^2^ and relatively elongate zeugopodia (Fig. 4) would indicate that they would have been quite capable of limb-driven propulsion in the water, especially through the use of their elongated hindlimbs. Rather than being a primarily tail driven swimmer as has been previously suggested^30^, mesosaurs could have used a combination of limbs and tail during aquatic locomotion, regardless of whether they were autotomizing their tails. Swim traces ascribed to mesosaurs have shown that while the tail was used in aquatic propulsion, the hind limbs would have also been used as a source of propulsion^45^. Overall, this suggests that, regardless of whether they could drop their tails or not, the limbs of mesosaurs would have played a larger role in aquatic locomotion than has previously been considered, something that warrants further investigation.

## Conclusions

Using numerous specimens, µCT data, and histological thin sections we confirm the presence of fracture planes on mesosaur caudal vertebrae. The presence of fracture planes indicates that mesosaurs had the potential to autotomize their tail, adding to the growing number of Palaeozoic tetrapods that had this feature, despite it classically being attributed to lepidosaurian diapsid reptiles. Furthermore, the potential for caudal autotomy in mesosaurs has implications for the swimming mode in the earliest aquatic amniotes. The presence of fracture planes combined with their limb anatomy suggests more of a reliance on limb driven propulsion than previously considered. However, the abundance of articulated mesosaur tails reveals that the presence of fracture planes in mesosaurs may be an evolutionary relic, as mesosaurs were restricted to an inland sea in which they had no known predators and may not have used the behaviour, suggesting that caudal autotomy evolved prior to their initial radiation into aquatic habitats.

## Material and Methods

### Fossil material

We examined several mesosaur specimens for the purpose of this study, these included representatives of all currently known mesosaur genera: *Stereosternum tumidum*, *Brazilosaurus sanpauloensis*, and *Mesosaurus tenuidens*. Sixty-three specimens were examined in total, the details of these specimens can be found in Table S1. Given that these three genera are monotypic, they will be referred to by their generic epithet for convenience.

### Heat map of fracture planes

Out of the 63 specimens with caudal vertebrae that we examined, 42 of them had their sacrum preserved, which allowed us to accurately identify the position of each caudal vertebrae. For each of these specimens, we identified on which caudal vertebrae fracture planes were visible (Table S2) and generated a heat map of the frequency of fracture plane presence on each caudal in R v3.6.1 (https://www.r-project.org). The heat map was then superimposed onto a schematic mesosaur tail (Fig. 1E) to facilitate visualization of the data.

### X-ray computed tomography

PIMUZ A/III 591 was characterized at the ID17 beamline of the European Synchrotron Radiation Facility (Grenoble, France), using propagation phase contrast synchrotron micro computed tomography. The beamline was set with a monochromatic beam of 120 keV (Laue bent double-crystal Si 111), 10 m of propagation between the sample and the detector; an indirect detector comprising a 2000 µm YAG scintillator, a ~0.3x magnification from a set of lenses, and a FReLoN 2k CCD camera. The resulting measured pixel size was 47.03 µm. Tomographic acquisition consisted of 4999 projections of 0.04 s each. As the specimen was larger than the field of view, an offset was applied on the centre of rotation, effectively increasing the reconstructed horizontal field of view by about 50%. Several acquisitions were performed to cover the specimen on the vertical axis. We used an overlap of ~50% between consecutive acquisitions to increase data quality by using a weighted average for low frequency. The high-frequency part of the overlap was averaged based on a defect map: we created a defect mask by thresholding a high pass filtered version of the flatfield image; when averaging the high frequency from the data with the specimen, we used the created map to exclude defects from the averaging. It resulted in a significant decrease of strong ring artefacts.

Tomographic reconstruction was performed using PyHST2^46^ with the single distance phase retrieval approach^47^. Additional post-processing comprised: 32 bits to 16 bits tiff conversion using the 0.002% saturation values from the 3D histogram produced by PyHST2; additional ring corrections^48^. Generated data were visualized using VG Studio Max 3.2 (https://www.volumegraphics.com) registered to J. Fröbisch at the Museum für Naturkunde Berlin.

### Histological thin sections

One thin section of a slightly damaged isolated mesosaur caudal vertebra was prepared at the Universität Bonn (IGPB R 624). The caudal vertebra was embedded in epoxy resin under vacuum and sectioned along the sagittal midline using a Buehler Isomet low-speed saw. Afterwards the section was then processed into a standard petrographic thin section 50–80 µm in thickness^49^. The histological thin section was imaged with a Leica DFC420 color camera mounted on a Leica DM2500LP polarizing microscope. Pictures were edited using the 2007 Leica IMAGE ACCESS EASYLAB 7 software (https://www.leica-microsystems.com).

## Supplementary information

Supplementary information.

Supplementary information.
