# Supplementary material for: Conflicting evidence for the use of caudal autotomy in mesosaurs
Source: Sci Rep. 2020 Apr 28;10:7184. doi: 10.1038/s41598-020-63625-0 (PMC7189235; doi:10.1038/s41598-020-63625-0)
Supplement: Supplementary file 1 — Supplementary information. [file 41598_2020_63625_MOESM1_ESM.docx]

**Conflicting evidence for the use of caudal autotomy in mesosaurs**

***Supplementary Information***

Mark J. MacDougall^1*^, Antoine Verrière^1^, Tanja Wintrich^2^, Aaron R. H. LeBlanc^3^, Vincent Fernandez^4, 5^ and Jörg Fröbisch^1, 6^

^1^Museum für Naturkunde, Leibniz-Institut für Evolutions- und Biodiversitätsforschung, Berlin, Germany;

^2^Rheinische Friedrich-Wilhelms-Universität Bonn, Bonn, Germany;

^3^University of Alberta, Edmonton, Canada;

^4^Natural History Museum London, London, UK;

^5^European Synchrotron Radiation Facility, Grenoble, France;

^6^Humboldt-Universität zu Berlin, Berlin, Germany.

*Corresponding author: mark.macdougall@mfn.berlin

Supplementary Table S1. List of specimens examined for this study.

Supplementary Table S2. Data used in the heatmap analysis.
